# Knowledge, Attitude, and Practice of Materiovigilance Among Healthcare Professionals at a Tertiary Care Teaching Hospital

**DOI:** 10.7759/cureus.64978

**Published:** 2024-07-20

**Authors:** Brijesh Sojitra, Chetna Patel, Sajal Pandya, Payal Virani, Paras Shah, Jaykumar Patel, Akash Shah

**Affiliations:** 1 Pharmacology, Government Medical College, Surat, Surat, IND; 2 Pharmacology, Pandit Dindayal Upadhyay Medical College, Rajkot, IND

**Keywords:** pharmacovigilance, health care professionals, materiovigilance, practice, attitude, knowledge

## Abstract

Objective

This study aimed to assess the knowledge, attitudes, and practices (KAP) of materiovigilance among healthcare professionals (HCPs) at a tertiary care teaching hospital in South Gujarat, India. Specifically, it sought to identify gaps in current understanding and reporting practices related to adverse events associated with medical devices.

Introduction

Materiovigilance, the systematic monitoring and assessment of adverse events related to medical devices, is crucial for ensuring patient safety and enhancing device performance. In India, the Materiovigilance Programme of India (MvPI) under the Ministry of Health and Family Welfare oversees the safe use of medical devices, integrating them with the pharmacovigilance framework. Despite these efforts, challenges persist in awareness, reporting practices, and the integration of materiovigilance among healthcare professionals. The knowledge, attitude, and practice (KAP) of these professionals are pivotal for effective adverse event reporting, but underreporting due to a lack of awareness, inadequate training, and perceived administrative burden remains a significant barrier. The study underscores the importance of training programs, improving reporting infrastructure, and fostering a safety culture within healthcare institutions to enhance the effectiveness of materiovigilance in India.

Methodology

An observational, cross-sectional study was conducted using a questionnaire-based approach. A total of 215 HCPs, including consultant doctors, resident doctors, and nursing staff, participated in the study. The questionnaire covered aspects of knowledge regarding the Materiovigilance Programme of India (MvPI), classification of medical devices, attitudes towards adverse event reporting, and actual reporting practices. Data collection was carried out electronically over the course of one month using Google Forms (Google LLC, Mountain View, California, United States).

Results

Among the participants, 135 (62.79%) correctly identified MvPI as the program for monitoring adverse events caused by medical devices. A majority of 188 (87.44%) understood that medical devices in India are classified based on a risk-based approach. Positive attitudes towards reporting adverse events were prevalent, with 202 (93.95%) acknowledging the potential for adverse events from medical devices and agreeing on the importance of reporting. However, a significant gap was noted between noticing adverse events (138 participants, 64.19%) and actual reporting (60 participants, 27.91%), indicating a need for improved reporting practices. Only 104 participants (48.37%) had participated in workshops or continuing medical education (CME) sessions on medical device safety.

Conclusion

The study reveals a strong foundation of knowledge and positive attitudes towards the materiovigilance among HCPs in South Gujarat. However, there is a notable discrepancy between awareness and actual reporting practices. To enhance the effectiveness of materiovigilance, interventions such as targeted educational programs and simplification of reporting procedures are recommended. These efforts are essential to ensure timely detection, reporting, and management of adverse events related to medical devices, thereby enhancing patient safety and overall healthcare quality.

## Introduction

Materiovigilance, the systematic monitoring and assessment of adverse events related to medical devices, is a critical component of healthcare aimed at ensuring patient safety and improving device performance. This practice involves the detection, assessment, reporting, and prevention of adverse effects or any other device-related issues [1].

In India, the Materiovigilance Programme of India (MvPI) was established under the aegis of the Ministry of Health and Family Welfare to oversee the safe use of medical devices [1]. The MvPI is part of a broader initiative to integrate materiovigilance into the existing pharmacovigilance framework, thereby ensuring comprehensive safety monitoring across pharmaceuticals and medical devices [2]. Despite these efforts, there remain substantial challenges in terms of awareness, reporting practices, and the overall integration of materiovigilance among healthcare professionals [3].

Healthcare professionals play a pivotal role in the success of materiovigilance systems. Their knowledge, attitude, and practice (KAP) towards adverse event reporting are essential for the timely identification and mitigation of risks associated with medical devices. Studies have shown that a significant proportion of adverse events remain underreported, which can hinder the effectiveness of materiovigilance programs [4]. Factors contributing to underreporting include lack of awareness, inadequate training, and perceived administrative burden [5,6].

This study aims to assess the knowledge, attitude, and practice (KAP) of materiovigilance among healthcare professionals at a tertiary care teaching hospital in South Gujarat. By examining the current state of KAP regarding materiovigilance, we seek to identify gaps and propose strategies to enhance the reporting and management of medical device-related adverse events. Previous studies have highlighted the importance of training and educational programs to improve the understanding and reporting of adverse events [7,8]. Additionally, enhancing the reporting infrastructure and simplifying reporting processes are critical for encouraging healthcare professionals to participate actively in materiovigilance [9,10].

The concept of materiovigilance encompasses a wide range of activities aimed at ensuring the safety and effectiveness of medical devices. These activities include post-market surveillance, adverse event reporting, and the continuous assessment of the benefit-risk balance of medical devices [3]. In India, the regulatory framework for medical devices has been evolving to keep pace with global standards. The Central Drugs Standard Control Organization (CDSCO) has been instrumental in developing guidelines and regulations to ensure the safe use of medical devices [2].

Despite these regulatory efforts, the success of materiovigilance programs largely depends on the active participation of healthcare professionals. The awareness and practice of materiovigilance among healthcare professionals are still in their nascent stages in many parts of India. The study found that while there is a general awareness of the importance of reporting adverse events, the actual practice of materiovigilance is often hindered by a lack of training and the perceived complexity of reporting procedures [11].

To address these challenges, targeted educational and training programs for healthcare professionals are crucial. These programs should focus on enhancing understanding of regulatory requirements for adverse event reporting, the importance of materiovigilance in patient safety, and practical aspects of reporting adverse events [7,8]. Integrating materiovigilance training into medical and nursing school curricula ensures new healthcare professionals are well-prepared to contribute to the system from the start of their careers [12].

Improving the reporting infrastructure is equally vital. Simplifying the reporting process significantly increases adverse event reporting rates [6]. This includes developing user-friendly reporting tools and providing clear reporting guidelines. Digital platforms and mobile applications enhance reporting efficiency and accessibility [10,13]. Fostering a safety culture within healthcare institutions is vital. Creating an environment where reporting adverse events is encouraged without fear of blame involves clear policies and procedures [3,14].

The findings from this study will provide valuable insights for policymakers, healthcare administrators, and educators to foster a culture of safety and vigilance in the use of medical devices. The ultimate goal is to ensure that the benefits of medical devices are maximized while minimizing any potential risks to patients [3]. By addressing the challenges in the current materiovigilance system and implementing effective strategies to enhance KAP among healthcare professionals, we can significantly improve the safety and effectiveness of medical devices in India.

## Materials and methods

Study design

This observational, cross-sectional, and non-interventional study adopted a questionnaire-based design to investigate the knowledge, attitudes, and practices (KAP) (Appendix 1) regarding materiovigilance among healthcare professionals (HCPs) at Government Medical College and New Civil Hospital, Surat.

Inclusion criteria

HCPs willing to participate in this study. These HCPs include consultant doctors, resident doctors, and nursing staff.

Exclusion criteria

Those who are not HCPs (consultant doctors, resident doctors, nursing staff) were excluded.

Sampling

The sample size was calculated using the Raosoft software (Raosoft, Inc., Seattle, Washington, United States) (http://www.raosoft.com/samplesize.html). The required sample size was estimated at the 95% confidence level, with an estimated 50% prevalence and a margin of error of ±5%. The required minimum sample size was determined to be 218. A random sampling technique was employed to recruit participants. This probability sampling technique was chosen due to its effectiveness in ensuring the availability and willingness of participants at a given time. Although the target sample size was 218, data collection was concluded at 215 participants, as this number was achieved within the predetermined data collection period of one month.

Data collection

A non-validated and structured questionnaire was developed based on the KAP framework related to materiovigilance. The questionnaire was inspired by a similar study conducted earlier [11]. The questionnaire was distributed electronically via email or WhatsApp (Meta Platforms, Inc., Menlo Park, California, United States) to the identified HCPs. This method allowed for efficient data collection while minimizing physical contact and time constraints.

Data analysis

Upon completion of data collection, responses were compiled and analyzed using Microsoft Excel version 2023 (Microsoft Corporation, Redmond, Washington, United States), which is freely available. Descriptive statistics were used to summarize the findings, including frequencies and percentages where applicable.

Timeline

Data collection spanned one month, followed by analysis and write-up, which were completed over one month; hence, a total of two months were spent conducting the study.

Ethical considerations

The study was conducted following approval from the Institutional Review Board (IRB) to ensure ethical standards were met. The study adhered to ethical guidelines to protect the rights and confidentiality of participants. Informed consent was obtained from all participants before they started the questionnaire.

## Results

Demographic details

A total of 215 healthcare professionals participated in the study, categorized by profession and department. The professional distribution included 47 consultant doctors/faculty (22%), 55 nursing staff (26%), and 113 resident doctors (53%).

**Figure 1:**
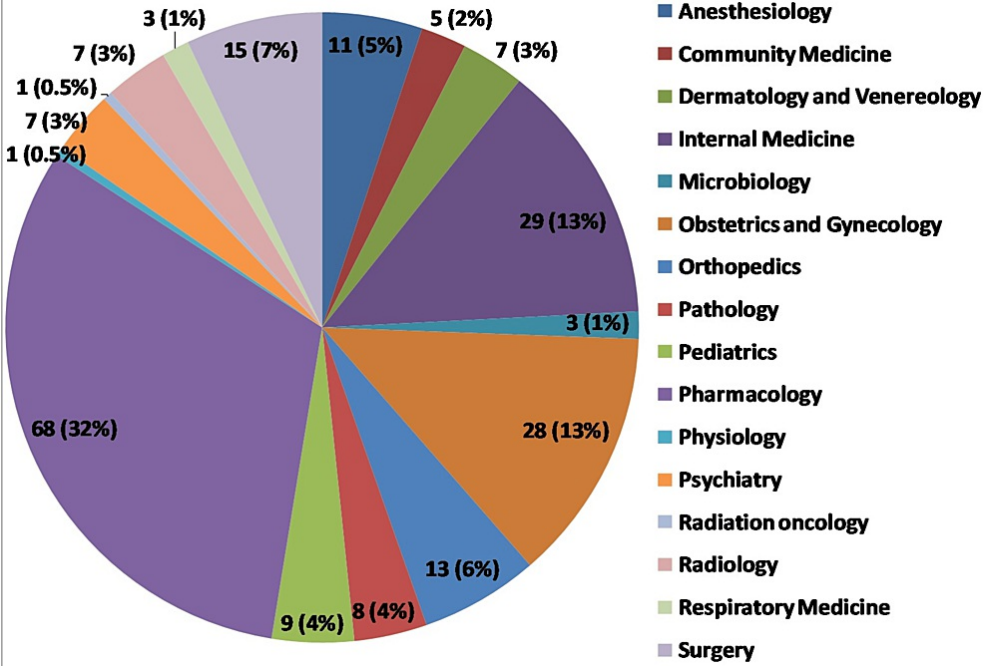
Departmental affiliation of participants

In terms of departmental affiliation, the largest group was from pharmacology with 68 participants (31.63%), followed by internal medicine with 29 participants (13.49%), and obstetrics and gynecology with 28 participants (13.02%). Other notable departments included orthopedics with 13 participants (6.05%), surgery with 15 participants (6.51%), and anesthesiology with 11 participants (5.12%). Smaller groups came from dermatology and venereology, psychiatry, radiology, and pediatrics, cumulatively comprising 30 participants (14%) of the total. Departments such as physiology, radiation oncology, and respiratory medicine each contributed fewer than two participants (less than 1%) (Figure. 1).

Knowledge-based questions

The majority, 135 (62.79%), identified the Materiovigilance Programme of India (MvPI) as the current program for monitoring adverse events caused by medical devices, while 44 (20.47%) believed all listed programs (PvPI, MvPI, and HvPI) were relevant. Most participants, 167 (77.67%), recognized that anyone with direct or indirect knowledge of a medical device adverse event can report it. Healthcare professionals alone were noted by 32 (14.88%), and a smaller fraction acknowledged manufacturers, importers, or distributors by five (2.33%) (Table 1).

**Table 1:** Shows the distribution of responses to the above question

Distribution of Responses to the Question: "Who can report adverse events caused by medical devices in India?"	Number of responses
Manufacturer/ importer/ distributor of medical devices	5 (2.33%)
Healthcare professionals	32 (14.88%)
Anyone with direct/ indirect knowledge of medical devices adverse event	11 (5.12%)
All of the three mentioned earlier	167 (77.67%)
Total	215 (100%)

A significant majority, 188 (87.44%), correctly identified that medical devices in India are classified based on a risk-based approach, considering factors like invasiveness and intended purpose. Other incorrect responses included cost, popularity, and manufacturing origin (Table 2).

**Table 2:** Shows the distribution of responses to the above question

Response to: "What criteria are used for the classification of medical devices into different categories (A, B, C, D) in India?"	Number of responses
The classification of medical devices in India is determined by the cost	10 (4.65%)
The classification of medical devices in India (A, B, C, D) relies on a risk-based approach	188 (87.44%)
In India, medical devices in India are categorized according to their popularity	12 (5.58%)
The classification of medical devices in India is solely based on their manufacturing origin	5 (2.32%)
Total	215 (100%)

A majority, 114 (53.02%), correctly identified non-invasive devices like thermometers as Category B devices. Other responses included intravenous infusion pumps, 44 (20.47%), surgical implants, 34 (15.81%), and MRI machines, 23 (10.70%).

The highest number of participants, 77 (35.81%), correctly noted that routine maintenance activities do not require reporting. Other common answers included scheduled software updates, 47 (21.86%), events related to employee training sessions, 50 (23.26%), and minor deviations from standard procedures, 41 (19.07%) (Table 3).

**Table 3:** Shows the distribution of responses to the above question

Responses to the Question: “Which event among the following does not require reporting?”	Number of responses
Routine maintenance activities on medical equipment typically do not require reporting as they are considered non-reportable events	77 (36%)
Minor deviations from standard operating procedures that have no impact on product quality or patient safety are events that need not be reported	41 (19%)
Scheduled software updates with no adverse impact on device functionality usually fall into the category of non-reportable events	47 (22%)
Events related to employee training sessions, if they do not affect product quality or patient safety, may not necessitate reporting	50 (23%)
Total	215 (100%)

Most participants, 170 (79.07%), correctly identified all given methods (medical device monitoring centers, the Indian Pharmacopoeia Commission (IPC) helpline, and email) as appropriate for reporting adverse events. Less common responses included individual methods such as reporting through medical device monitoring centers by 24 (11.16%) and via email by 12 (6%) (Table 4).

**Table 4:** Shows the distribution of responses to the above question

Distribution of Responses to the Question: “What is the appropriate way to report an adverse event resulting from a medical device?”	Number of responses
At medical device monitoring centers (MDMC) after filling the MDAE reporting form	24 (11%)
Via calling IPC helpline number 1800-180-3024	9 (4%)
By sending scanned filled copy of MDAE reporting form via mail to mvpi.ipcindia@gmail.com	12 (6%)
All of the above	170 (79%)
Total	215 (100%)

Attitude-based questions

A vast majority, 202 (93.95%), acknowledged that medical devices could induce adverse events in patients. Most participants, 201 (93.49%), believed it was essential to report adverse events related to medical devices. Similarly, 202 (93.95%) agreed that it is the responsibility of healthcare professionals to report adverse events. An overwhelming majority, 201 (93.49%), agreed that reporting adverse events would improve patient safety. A majority of 176 (81.86%) supported the encouragement of laypersons to report adverse events related to medical devices.

Practice-based questions

More than half of the participants, 138 (64.19%), reported noticing adverse events related to medical devices during their professional practice. However, only 60 (27.91%) had actually reported these events, indicating a gap between observation and reporting. About half, 114 (53.02%), participants actively monitored patients for potential adverse outcomes from implanted devices beyond the recovery period. Similarly, 118 (54.88%) took feedback from patients regarding any untoward events post-implantation. A majority, 135 (62.79%), were familiar with the adverse event reporting form for medical devices created by the Central Drugs Standard Control Organization (CDSCO). Indicating a need for increased educational efforts in this area, 104 (48.37%) had participated in workshops or continuing medical education (CME) sessions specifically addressing the safety of medical devices.

## Discussion

The study's findings indicate a robust awareness among healthcare professionals regarding the current program for monitoring adverse events caused by medical devices, with 135 (62.79%) correctly identifying the Medical Device Vigilance Programme of India (MvPI) as the relevant program [1]. This aligns with national efforts to increase awareness of materiovigilance through initiatives and training programs [3].

The understanding of who can report adverse events is also noteworthy. A significant 167 (77.67%) of participants recognized that anyone with direct or indirect knowledge of an adverse event can report it. This broad perspective is essential for comprehensive adverse event capture and reflects the guidelines of the UMC [15].

When it comes to the classification of medical devices, a large majority, 188 (87.44%), correctly identified that devices are classified based on a risk-based approach. This knowledge is critical as it influences the monitoring and reporting processes [7,16]. Furthermore, understanding the categorization of medical devices, such as recognizing non-invasive devices like thermometers as Category B, is crucial for appropriate monitoring and reporting [2].

A vast majority, 202 (93.95%), acknowledged that medical devices could induce adverse events, and a similar proportion, 201 (93.49%), believed in the importance of reporting these events [5]. This positive attitude towards reporting aligns with findings from previous studies emphasizing that proactive reporting is vital for patient safety [17].

Additionally, most participants, 202 (93.95%), agreed that it is the responsibility of healthcare professionals to report adverse events, reflecting a strong sense of professional duty. This attitude is consistent with global perspectives on pharmacovigilance, where healthcare professionals play a crucial role in monitoring and reporting adverse events [18].

Encouragingly, a majority of 176 (81.86%) supported the idea of encouraging laypersons to report adverse events. This inclusive approach can enhance the reporting system's comprehensiveness and is in line with the World Health Organization's recommendations for patient and public involvement in pharmacovigilance [19].

Despite the high level of awareness and positive attitudes, there is a noticeable gap between the observation and reporting of adverse events. While 138 (64.19%) of participants reported noticing adverse events, only 60 (27.91%) actually reported them. This discrepancy highlights a significant area for improvement. Barriers to reporting, such as lack of time, awareness, or fear of legal consequences, have been documented in various studies and need to be addressed through targeted interventions [6].

Moreover, only 114 (53.02%) of participants actively monitored patients for potential adverse outcomes post-implantation, and 118 (54.88%) took feedback from patients regarding any untoward events. These practices are essential for the early detection and management of adverse events and should be emphasized in training programs [9].

Familiarity with the adverse event reporting form for medical devices was observed in 135 (62.79%) of participants, indicating a good level of procedural knowledge. However, participation in workshops or CME sessions specifically addressing medical device safety was reported by only 104 (48.37%) of the participants. Increasing participation in such educational initiatives could significantly enhance reporting practices [20].

This study has several limitations. First, the use of a non-validated questionnaire may affect the reliability of the data. Second, the cross-sectional design captures only a snapshot in time and may not reflect changes in knowledge, attitudes, or practices over time. Third, the reliance on self-reported data could introduce bias, as participants may overstate their knowledge or practices. Additionally, the study was conducted in a single tertiary care teaching hospital in South Gujarat, limiting the generalizability of the findings to other regions or types of healthcare institutions. Finally, the response rate and sample size, although sufficient for this study, might not adequately represent the entire population of healthcare professionals.

## Conclusions

The study highlights a high level of knowledge and positive attitudes towards materiovigilance among healthcare professionals at a tertiary care teaching hospital in South Gujarat. However, the gap between the observation and reporting of adverse events suggests a need for improved training and support systems. Enhancing awareness through workshops and continuous medical education, along with simplifying the reporting process, could foster a more effective materiovigilance system. Ensuring the safety of medical devices through vigilant reporting and monitoring is crucial for patient safety and aligns with national and international efforts to improve healthcare outcomes.

## Appendices

Appendix 1

Knowledge-Based Questions

1). What is the current program in India for monitoring adverse events caused by medical devices?

A) PvPI

B) MvPI

C) HvPI

D) All of the above

2). Out of the following, who can report adverse events caused by medical devices in India?

A) Manufacturer/ importer/ distributor of medical devices

B) Healthcare professionals

C) Anyone with direct/indirect knowledge of medical devices adverse event

D) All of the above

3). What criteria are used for the classification of medical devices into different categories (A, B, C, D) in India?

A) The classification of medical devices in India is determined by the cost

B) The classification of medical devices in India (A, B, C, D) relies on a risk-based approach, evaluating factors like invasiveness and intended purpose

C) In India, medical devices in India are categorized according to their popularity

D) The classification of medical devices in India is solely based on their manufacturing origin

4). Identify the medical device that is classified under category B from the following options:

A) MRI machines, due to their complexity and potential risks, fall under category B in India

B) Non-invasive devices like thermometers fall into category B, indicating a low to moderate risk level in the Indian classification system

C) Intravenous infusion pumps, being invasive devices, are classified as category B medical devices in India

D) Surgical implants, such as hip prostheses, are considered category B devices in the Indian classification system

5). Which event among the following does not require reporting?

A) Routine maintenance activities on medical equipment typically do not require reporting as they are considered non-reportable events

B) Minor deviations from standard operating procedures that have no impact on product quality or patient safety are events that need not be reported

C) Scheduled software updates with no adverse impact on device functionality usually fall into the category of non-reportable events

D) Events related to employee training sessions, if they do not affect product quality or patient safety, may not necessitate reporting

6). What is the appropriate way to report an adverse event resulting from a medical device?

A) At Medical Device Monitoring Centers (MDMC) after filling out the MDAE reporting form

B) Via calling the IPC helpline number 1800-180-3024

C) By sending scanned, filled-out copy of the MDAE reporting form via mail to mvpi.ipcindia@gmail.com

D) All of the above

Attitude-Based Questions

1). Do you think that medical devices have the potential to induce adverse events in patients?

                YES_______                      NO_______

2). Do you believe it is essential to report any adverse events linked to medical devices?

                YES_______                      NO_______

3). Do you agree that it is the responsibility of doctors to report adverse events resulting from medical devices?

                YES_______                      NO_______

4). Do you concur that the reporting of adverse events will contribute to the improvement of patient safety?

                YES_______                      NO_______

5). Should the reporting of adverse events due to medical devices by laypersons be encouraged in India?

                YES_______                      NO_______

Practice-Based Questions

1). Have you ever noticed any adverse events related to medical devices during your professional practice?

                YES_______                      NO_______

2). Have you ever reported any adverse events related to medical devices during your professional practice?

                YES_______                      NO_______

3). Do you actively observe patients for potential adverse outcomes from implanted devices beyond the recovery period?

                YES________                    NO_______

4). Do you take feedback from patients regarding any untoward events after the implantation of a device?

                YES_______                      NO_______

5). Are you familiar with the adverse event reporting form for medical devices created by CDSCO?

                 YES_______                     NO_______

6). Have you ever participated in any workshop or CME session specifically addressing the safety of medical devices?

                YES_______                      NO_______
